# Probability-Based Recognition Framework for Underwater Landmarks Using Sonar Images †

**DOI:** 10.3390/s17091953

**Published:** 2017-08-24

**Authors:** Yeongjun Lee, Jinwoo Choi, Nak Yong Ko, Hyun-Taek Choi

**Affiliations:** 1 Marine Robotics Laboratory, Korea Research Institute of Ships and Ocean Engineering, Daejeon 34103, Korea; leeyeongjun@kriso.re.kr (Y.L.); jwchoi@kriso.re.kr (J.C.); 2 Department of Electronics Engineering, Chosun University, Gwangju 61452, Korea; nyko@chosun.ac.kr

**Keywords:** underwater object recognition, framework, artificial landmark, imaging sonar, robot intelligence

## Abstract

This paper proposes a probability-based framework for recognizing underwater landmarks using sonar images. Current recognition methods use a single image, which does not provide reliable results because of weaknesses of the sonar image such as unstable acoustic source, many speckle noises, low resolution images, single channel image, and so on. However, using consecutive sonar images, if the status—i.e., the existence and identity (or name)—of an object is continuously evaluated by a stochastic method, the result of the recognition method is available for calculating the uncertainty, and it is more suitable for various applications. Our proposed framework consists of three steps: (1) candidate selection, (2) continuity evaluation, and (3) Bayesian feature estimation. Two probability methods—particle filtering and Bayesian feature estimation—are used to repeatedly estimate the continuity and feature of objects in consecutive images. Thus, the status of the object is repeatedly predicted and updated by a stochastic method. Furthermore, we develop an artificial landmark to increase detectability by an imaging sonar, which we apply to the characteristics of acoustic waves, such as instability and reflection depending on the roughness of the reflector surface. The proposed method is verified by conducting basin experiments, and the results are presented.

## 1. Introduction

Object recognition using visual sensors is one of the most important topics in autonomous robotic systems. The recognized objects or features can be used as landmarks to achieve environmental recognition and autonomous navigation. Similarly, in the underwater vision research area, several methods using optical images have also been applied to the underwater recognition and navigation [1,2,3,4,5,6]. Unfortunately, underwater optical images can be seriously affected by environmental conditions such as turbidity and illuminations. These environmental conditions make it difficult for underwater vehicles to use optical images. Alternatively, imaging sonars that use acoustic signals are utilized in the underwater vision research area. An imaging sonar is not affected by illuminations and has long-distance visibility. Due to these characteristics, imaging sonars have been widely used in various studies [7,8,9,10,11,12,13,14]. However, accurate object recognition is still difficult because of the practical performance of sonars such as the low resolution image, unstable acoustic source, many speckle noise, and only gray scale image [15,16].

To overcome these challenges, this paper proposes a probability-based framework for underwater landmark recognition using an imaging sonar. Current recognition methods use a single sonar image that does not provide reliable results due to the weaknesses of sonar images as mentioned above. On the other hand, if an object exists continuously in consecutive images and we can evaluate its features using a stochastic method, it is possible to calculate, with certainty, the features of the object; as a result, the reliability of recognition increases. To achieve this, we design a recognition framework that consists of three steps: (1) candidate selection, (2) continuity evaluation and (3) Bayesian feature estimation. Two probability methods, particle filtering and Bayesian feature estimation, are used to evaluate the continuity and features of an object in a series of images. In addition, to increase detectability by an imaging sonar, we develop an artificial landmark that takes into account the characteristics of acoustic waves related to reflections.

The proposed method results in a well-structured framework of underwater object recognition using imaging sonar. The proposed method has several advantages as follows. First, the proposed improves the visibility of landmark in sonar images by adopting a specialized artificial landmark. Second, a particle filter can perform an effective evaluation of the continuity of target object in consecutive images. Finally, a probabilistic framework based on Bayesian feature estimation provides reliable object recognition results even with noisy sonar images.

The remainder of this paper is organized as follows: Section 2 summarizes related work on imaging sonars in underwater environments; Section 3 describes the proposed probability-based underwater object recognition using imaging sonar; Section 4 presents experimental results in a basin and Section 5 concludes the paper.

## 2. Background

### 2.1. Previous Work

Many studies have been conducted to apply image registration and object recognition using sonar images to areas such as robot navigation. For example, Hurtos et al. conducted a study on Fourier-based registration for sonar mosaicing [17]. The authors employed a phase correlation based method that estimates the rotation and translation between consecutive frames. After the global alignment, the result not only present the mosaic image but also the path of sonar movement. Similarly, another study [18] proposes a Normal Distribution Transform (NDT) algorithm for the registration used for the hull inspection of Autonomous Underwater Vehicle (AUV). Fallon et al. develop a sonar-based Simultaneous Localization And Mapping (SLAM) using natural features on the seabed [19]. When the AUV, such as REMUS 100, moves around the sea, the vehicle position is estimated using natural features from sonar with dead-reckoning data. Furthermore, acoustic shadows have been used to recognize an underwater object [20]. The sonar emits an acoustic wave to the objects; however, the acoustic wave cannot reach the back of an object and this region is dark in the sonar image. Hence, the descriptor of the object is used because the shape differs for each object. In another study on object recognition, Cho et al. propose a cross correlation-based object detection method using sonar-beam profiles [21]. This method quickly recognizes the presence of an object by using the difference of intensity in consecutive frames. A number of these studies require a descriptor, which represents a feature of the object or a point in the sonar image. However, it remains a challenge to define a descriptor that has robustness and repeatability, owing to the weaknesses of sonar images.

### 2.2. Artificial Landmarks and Imaging Sonar

Considering the constraints on achievable quality of sonar images, the only way to obtain stable and accurate information for navigation is by using well-designed artificial landmarks. Sonar measures the travel time and intensity of a reflected acoustic wave in order to identify an underwater object. Thus, we can obtain clearer sonar images if the surface of the object has different characteristics making contrasting reflections. Various experiments have been conducted to find appropriate materials to achieve a sharp contrast; a two-dimensional artificial landmark was designed using gel-stone on an aluminum plate. Gel-stone reflects much of the transmitting wave; moreover, it is easy to handle. On the contrary, an aluminum plate reflects less of the transmitting wave and it has enough weight to be placed on the sea bottom.

In the early stage of designing the landmarks, we considered a letter type that has sufficient information not only for the landmark but also for being recognized by a human. However, even though a letter is drawn using the proposed method, line features of the letter are not very clear owing to the limitation of sonar technologies; as a result, lines are considered as an inappropriate feature.

**Figure 1:**
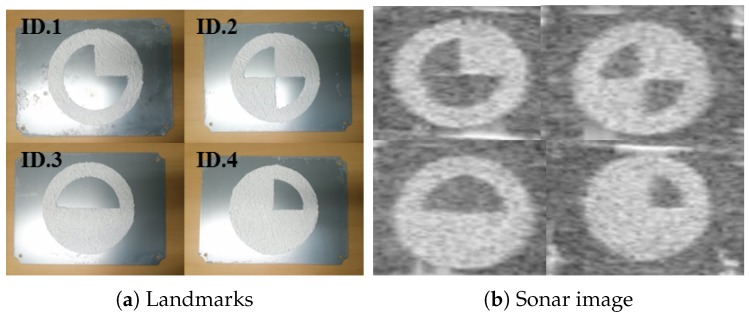
Developed artificial landmarks for imaging sonar: landmarks are manufactured using the differences in acoustic reflection based on their surface roughness.

Therefore, we developed circular-shaped landmarks combined with four different symbols designed for the inside as shown in Figure 1. Using the sonar parameters provided by a sonar, a circular-shape can be easily modeled to implement an algorithm for detecting four different landmarks. Furthermore, it can be differentiated from natural objects and detected regardless of direction. On the other hand, four different symbols have robustness against low quality images and environment noise and disturbances because they use an area-based feature. To realize the omni-directional characteristic of landmarks, they are placed orthogonal to the vertical axis, which is not an unreasonable constraint because a landmark could be placed in accordance with any human intention.

In spite of a well-designed landmark composed of specially selected materials, exteriors, and area-based features, the features in a single sonar image frame are not sufficient to identify the landmark because of various noises and disturbances from the real underwater environment. Therefore, consecutive sonar images should be considered within a probability-based framework to derive stable information for navigating an underwater robot.

## 3. Probability-Based Recognition Framework

The proposed method can effectively recognize underwater landmarks principally because the probabilistic approach of Bayesian estimation can provide reliable results of object recognition even when sonar images are affected by noisy data.

### 3.1. Overall Framework

**Figure 2:**
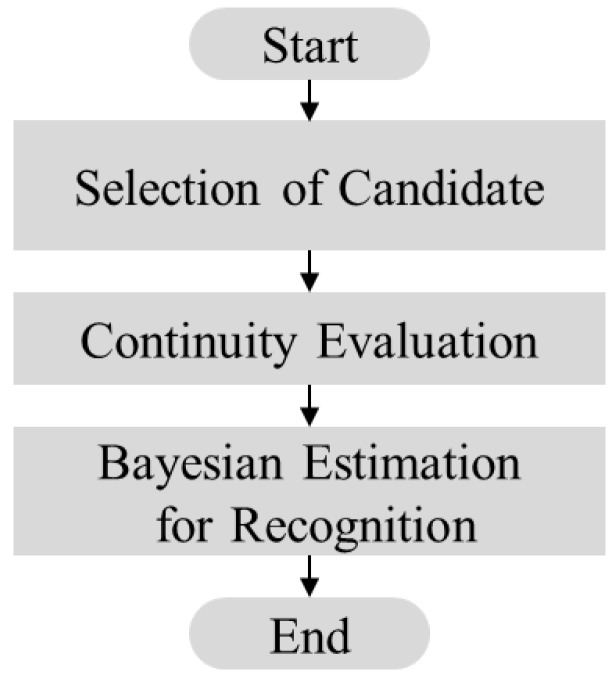
Proposed framework of probability-based recognition.

**Figure 3:**
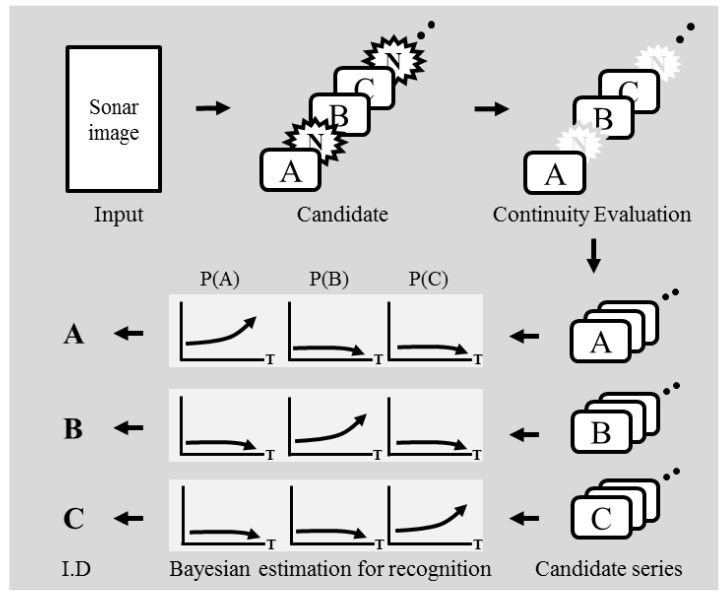
Flowchart of probability-based recognition framework.

The proposed method is achieved by a framework comprising three processes. First, candidates of target objects are acquired from sonar images using a predefined landmark model. Second, a particle filter is used to evaluate the continuity of the acquired candidates in consecutive images. Lastly, a probabilistic approach based on Bayesian feature estimation is used to recognize and to identify the detected objects. Figure 2 and Figure 3 depict an overview of the proposed probability-based recognition framework and a flowchart of the algorithm, respectively.

### 3.2. Selection of Candidate

Generally, a series of image-processing algorithms is applied to an entire image to detect landmarks. Although this may be an effective technique for optical images, it is not practical for acoustic images because of the non-uniform characteristics of local regions in sonar images, due to the reasons mentioned earlier. To overcome this, we first attempt to find objects similar to a landmark from the entire image, and only the selected objects are transferred to the next step.

**Figure 4:**
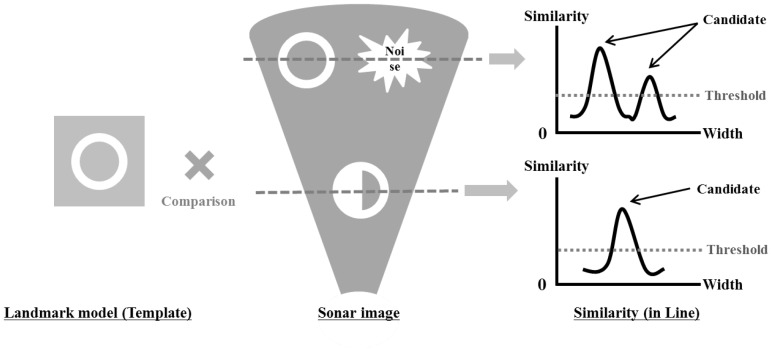
Process of selection of candidate: this is similar to the conventional template matching method. A landmark model is compared with the sonar image and the high similarity region is to be a candidate.

**Figure 5:**
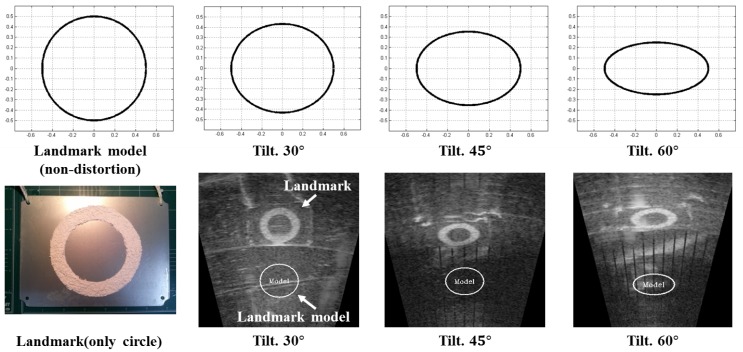
Landmark models according to tilt (downward) of sonar.

**Figure 6:**
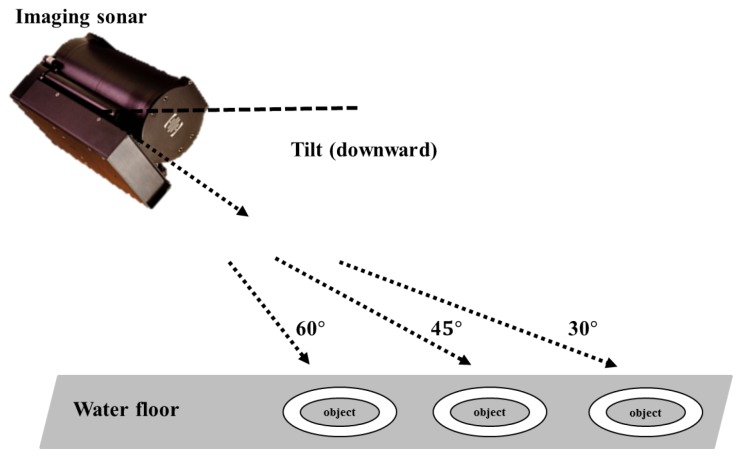
Sonar set-up for acquiring an image: the object should be placed at the bottom of the sonar for a good image.

The selected objects are referred to as candidates in this step. The candidates are obtained through comparison with a landmark model as shown in Figure 4. This process is similar to the conventional template matching method used in the image processing technique. A landmark model is compared with the sonar image, and the point with the high similarity is selected as the candidate. The landmark model can have various forms depending on the landmark. We use an artificial landmark having a circular shape with various inner fan shapes. Hence, a circle is used to model a landmark in this study. The landmark model is presented in Figure 5. The landmark model should be changed according to the sonar parameters related to sonar pose and range of view because the sonar must look down towards the objects for acquiring the image as shown in Figure 6 and it can change its range of view. For this reason, these conditions cause distortions of object in terms of vertical deformation and the size in sonar images. Hence, considering these two conditions, the landmark model is calculated using the following equations. Equations (1) and (2) represent the deformation due to tilt (downward) of sonar and the scale considering the range of view, respectively.

The circular outline of the landmark on the *x*- and *y*-axis is denoted by $x_{c}$, $y_{c}$ and the deformed line related to the tilt angle ψ is denoted by $x_{d}$, $y_{d}$ where the unit is metric. The resultant landmark model in image plane is *u*, *v*, where the unit is *pixel*. *r* is a range of view in meter and the constant 512 is the height of the image:

(1) $$\left[\begin{array}{c} x_{d}\\ y_{d} \end{array}\right]=\left[\begin{array}{cc} 1 & 0\\ 0 & cos\;\psi \end{array}\right]\cdot \left[\begin{array}{c} x_{c}\\ y_{c} \end{array}\right],$$

(2) $$\left[\begin{array}{c} u\\ v \end{array}\right]=\left[\begin{array}{cc} 1/(r/512) & 0\\ 0 & 1/(r/512) \end{array}\right]\cdot \left[\begin{array}{c} x_{d}\\ y_{d} \end{array}\right].$$

**Figure 7:**
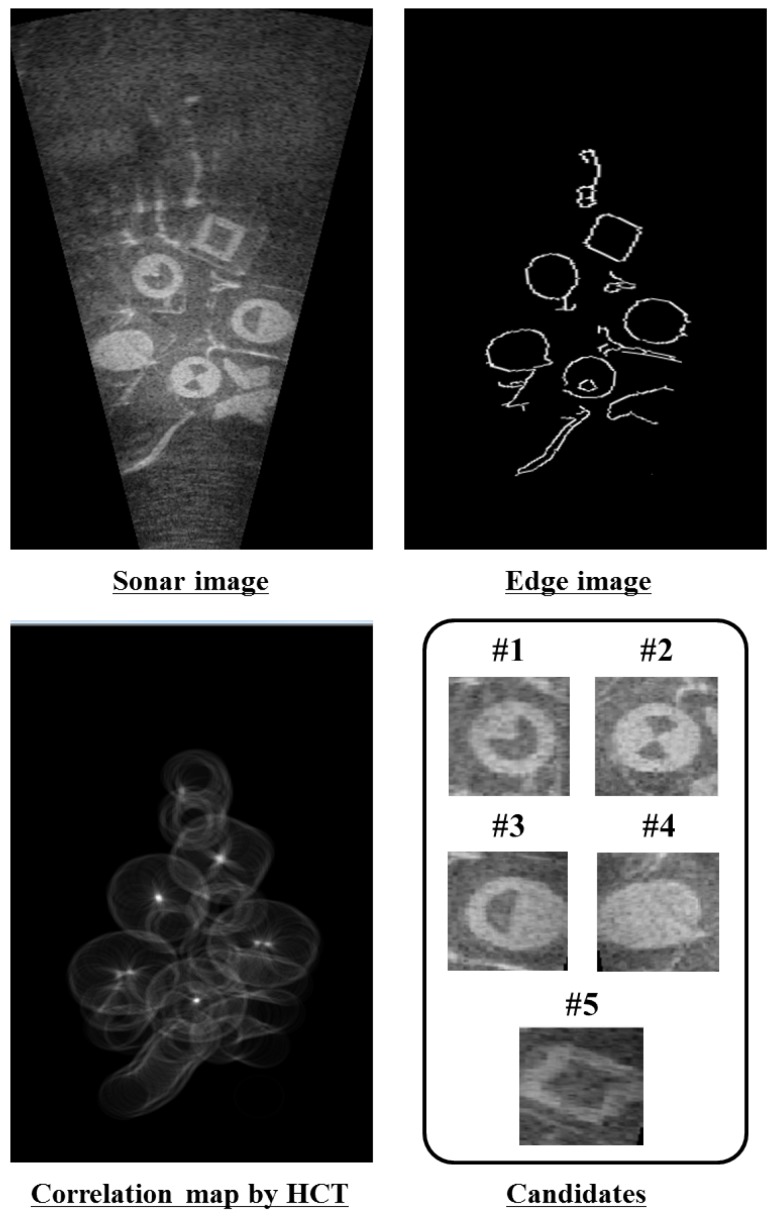
Experimental example of selection of candidates.

To select the candidates that have a circular-shape in a sonar image, the Hough circle transform technique is adopted [22]. It requires a correlation map as shown in Figure 7. The correlation map is calculated by comparing the landmark model and an edge image. It represents a possibility that a landmark exists in a recent sonar image. Thus, the high score region in the correlation map is considered as candidates. If all correlation values are lower than the number of pixels of the landmark model, we determine that no candidates exist in the current image.

### 3.3. Continuity Evaluation

The candidates selected in the current image have no connectivity to the previous image. Only if we know the series of information for any candidate, consecutive stochastic recognition will be possible because its inputs as measurements are necessary to obtain continuity. To solve this problem, particle filter method is applied to evaluate the continuity of candidates in a series of images.

**Figure 8:**
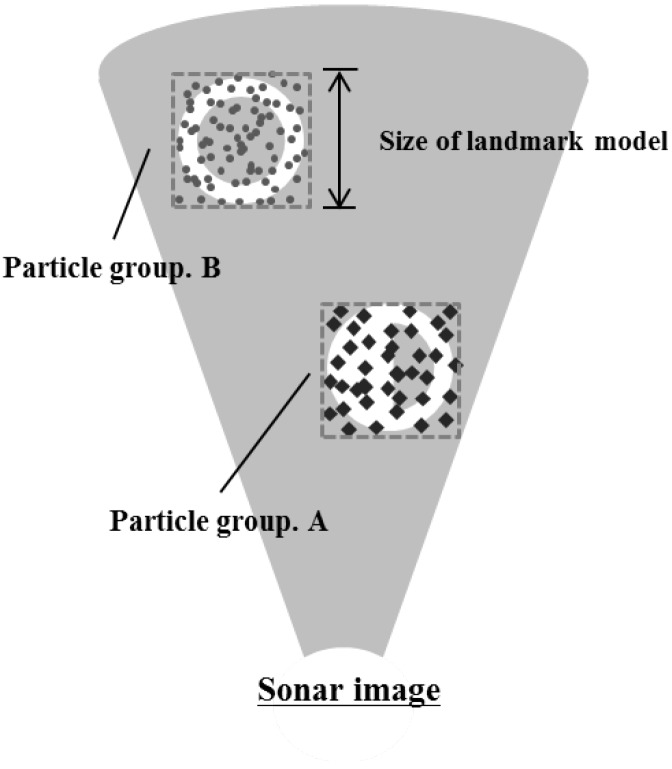
Initialization of particle groups.

Particle filter is applied to each likely candidate. In addition, 1000 to 2000 particles are randomly scattered around the position of each likely candidate and it is designated as a particle group as shown in Figure 8. In the general prediction phase of particle filter, the system is predicted by motion model using dead-reckoning data. However, the adapted particle filter uses only a sonar image. For this reason, we adopted an uncertainty motion function as Equation (3). $L_{t,i}$ is a predicted particle position at *t*, and $L_{t-1,i}$ is the previous particle position. randn(σ) is a function to determine uncertainty depending on standard normal distribution and σ is the parameter value for movement of candidate. σ was set to 10pixels in this study. It can be considered reasonable because the angle of view is too small, i.e., $28.8^{\circ }$ and the sonar moves slowly:

(3) $$L_{t,i}=L_{t-1,i}+randn\left(\sigma \right).$$

After the prediction, the belief of each particle is updated by using the sensor model as in Equation (4). As in the selection of candidates step, the extracted edge image and landmark model are used to calculate the belief. In other words, the belief of particle is defined to check the existence of similar shapes as that of the landmark, around the particles. *P* is a belief of particles, $p_{hit}$ is probability of existence of a circle from correlation map and $p_{rand}$ is equally given probability. $z_{hit}$ and $z_{rand}$ are the weight values for each probability:

(4) $$P\;=z_{hit}\cdot P_{hit}+z_{rand}\cdot P_{rand}.$$

**Figure 9:**
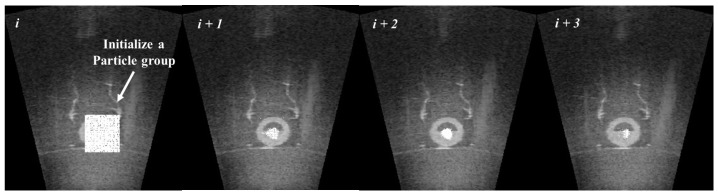
Convergence of particles at the candidate.

Figure 9 shows a simple result of particle filter. Particles find a landmark and congregate densely to the center of the landmark in a series of images.

A particle filter not only evaluates the continuity of candidates but also eliminates a fault candidate. The particles or particle group on the landmark will be congregated as mentioned above, but the particles on the fault candidate like noise will be scattered continuously. Therefore, if the standard deviation of a particle group is larger than 25% of the size of the landmark model five times, we decide that the candidate is not the object of interest and delete the particle group. As a result, filtered candidates are the output of the continuity evaluation step. A mean position of particle group is offered to the next step.

### 3.4. Bayesian Feature Estimation for Recognition

To recognize a landmark, we use a Bayesian feature estimation method. This method estimates probabilities of features of an object using a method based on Bayes’ rule and the feature refers to the object name as ID.1, ID.2, ID.3, ID.4. In this step, the candidate is to be a landmark we want to identify and each landmark is evaluated based on the probabilities of features in a series of images. As a result, the high probability of feature is considered as its ID.

Table 1 shows the notations for the calculation using the Bayesian feature estimation method.

#### 3.4.1. Estimation Procedure

An estimation procedure consists of three steps: prediction, update by motion, and update by measurement. Algorithm 1 outlines the procedure. The procedure estimates the probability $p(x_{t(i)}\left(m\right)=f_{k})$ using the measurement model and the measurement information.

**Algorithm 1** Bayesian feature estimation procedure.1:**procedure** ($p(x_{t(i-1)}\left(m\right)=f_{k})$, $p(l_{t(i)}\left(m\right)=l_{t(i-1)}\left(j\right))$, $p(z_{t(i)}\left(m\right)=f_{j}|x_{t(i)}\left(m\right)=f_{k})$, $p\left(z_{t(i)}\left(m\right)=f_{j}\right),j=1,2,\cdots ,F_{feature}$)2:  Predict $\bar{p}\left(x_{t(i)}\left(m\right)=f_{k}\right)$ based on the $p(x_{t(i-1)}\left(m\right)=f_{k})$ using the motion model3:  Find $p(x_{t(i)}\left(m\right)=f_{k}|z_{t(i)}\left(m\right)=f_{j})$ using the measurement model4:  Update $\bar{p}\left(x_{t(i)}\left(m\right)=f_{k}\right)$ to $p(x_{t(i)}\left(m\right)=f_{k})$ using measurement5:  Return $p(x_{t(i)}\left(m\right)=f_{k})$6:**end procedure**

Line 2 predicts the probability $\bar{p}\left(x_{t(i)}\left(m\right)=f_{k}\right)$ that the landmark *m* corresponds to the feature $f_{k}$ before there is information from measurement. Line 2 is described in detail as Equation (5). $p(l_{t(i)}\left(m\right)=l_{t(i-1)}\left(j\right))$ is called the motion model. It is the probability that the landmark *j* at time $t_{\left(i-1\right)}$ corresponds to the landmark *m* at time $t_{\left(i\right)}$. It can be calculated using an inverse distance between each landmark:

(5) $$\bar{p}\left(x_{t(i)}\left(m\right)=f_{k}\right)=\sum_{j=1}^{M_{t(i-1)}}p\left(x_{t(i-1)}\left(j\right)=f_{k}\right)\cdot p\left(l_{t(i)}\left(m\right)=l_{t(i-1)}\left(j\right)\right).$$

Line 3 in Algorithm 1 shows the calculation of the probability that the detected landmark *m* corresponds to the feature *k* provided that the measurement assumes that the landmark has the feature *j*. Line 3 is explained in detail in Algorithm 2. Algorithm 2 shows two major steps. The first step calculates the conditional probability using Equation (6). Equation (6) uses the measurement model $p(z_{t(i)}\left(m\right)=f_{j}|x_{t(i)}\left(m\right)=f_{k})$. The measurement model $p(z_{t(i)}\left(m\right)=f_{j}|x_{t(i)}\left(m\right)=f_{k})$ describes the probability that the feature $f_{k}$ is recognized as the feature $f_{j}$ by the measurement system. It is determined by the performance and properties of the measurement system and is given a priori. The next step is the normalization of the conditional probability using Equation (7). The probability given in Equation (6) is divided by the normalizer $\eta_{f_{j}}$ to make the total probability theorem work for the conditional probability.

**Algorithm 2** Application of measurement model.1:η=12:**for** all $l_{t(i)}\left(m\right),m=1,2,\cdots ,M_{t(i)}$ **do**3:  **for** all $f_{k}$, $k=1,2,\cdots ,F_{feature}$ **do**4:    **for** all $f_{j}$, $j=1,2,\cdots ,F_{feature}$ **do**5:(6)$$\;p\left(x_{t(i)}\left(m\right)=f_{k}|z_{t(i)}\left(m\right)=f_{j}\right)=\frac{1}{\eta }\;p\left(z_{t(i)}\left(m\right)=f_{j}|x_{t(i)}\left(m\right)=f_{k}\right)\cdot p\left(x_{t(i)}\left(m\right)=f_{k}\right)$$6:    **end for**7:  **end for**8:  **for** all $f_{j}$, $j=1,2,\cdots ,F_{feature}$ **do**9:    $\eta_{f_{j}}=\sum_{k=1}^{F_{feature}}\hat{p}\left(x_{t(i)}\left(m\right)=f_{k}\right)|z_{t(i)}\left(m\right)=f_{j})$10:  **end for**11:  **for** all $f_{k},k=1,2,\cdots ,F_{feature}$ **do**12:    **for** all $f_{j},j=1,2,\cdots ,F_{feature}$ **do**13:(7)$$\;p\left(x_{t(i)}\left(m\right)=f_{k}\right)|z_{t(i)}\left(m\right)=f_{j})=\frac{1}{\eta_{f_{j}}}\hat{p}\left(x_{t(i)}\left(m\right)=f_{k}|z_{t(i)}\left(m\right)=f_{j}\right)$$14:   **end for**15:  **end for**16:**end for**

Line 4 in Algorithm 1 updates the predicted probability $\bar{p}\left(x_{t(i)}\left(m\right)=f_{k}\right)$ using the measurement information $p\left(z_{t(i)}\left(m\right)=f_{j}\right),j=1,2,\cdots ,F_{feature}$. The measurement update uses Equation (8):

(8) $$p\left(x_{t(i)}\left(m\right)=f_{k}\right)=\sum_{j=1}^{F_{f}}p\left(x_{t(i)}\left(m\right)=f_{k}|z_{t(i)}\left(m\right)=f_{k}\right)\cdot p\left(z_{t(i)}\left(m\right)=f_{j}\right).$$

Throughout the procedure, the motion model $p(l_{t(i)}\left(m\right)=l_{t(i-1)}\left(j\right))$ and measurement model $p(z_{t(i)}\left(m\right)=f_{j}|x_{t(i)}\left(m\right)=f_{k})$ are to be given a priori. The measurement $p\left(z_{t(i)}\left(m\right)=f_{j}\right),j=1,2,\cdots ,F_{feature}$ is provided by a measurement system that uses an image processing approach for feature identification of every detected landmark.

#### 3.4.2. Measurement System

**Figure 10:**
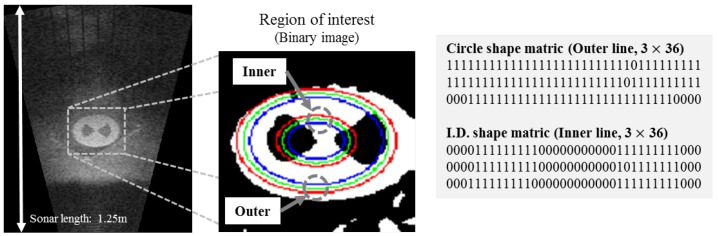
Example of shape matrices indentification (ID.2).

To get the measurement from a landmark, the shape matrices identification (SMI) method is applied to our study [23]. This method describes an area which is triangular, square, circular and any pattern. An information of area is expressed with the matrices called shape matrices. Figure 10 is a simple example, whose result is a shape matrix applying to landmark ID.2. As shown in the binary image in Figure 10, three outer lines evaluate a circle. It means that the landmark has the circular shape. In addition, three inner lines that check ID Ideal inner shape matrices of each ID are $S_{ID_{1}}=\left[IOOO\right]$, $S_{ID_{2}}=\left[IOIO\right]$, $S_{ID_{3}}=\left[IIOO\right]$, $S_{ID_{4}}=\left[IIIO\right]$, (I=3×9(all element is1),O=3×9(all element is0)). We calculate a similarity by comparison with an extracted shape matrix from an image and ideal inner shape matrices of each ID, and then normalize a similarity. The normalized similarity is used for a measurement in the updated state in the Bayesian feature estimation step. In addition, the average of the number of measurements under stable condition is a measurement model. However, there are differences by a range of views because the size of the landmark changed due to the field of view. We use an imaging sonar, DIDSON (Soundmetrics, Bellevue, WA, USA), and it can change a range of view to 1.25 m, 2.50 m, 5.0 m and 10.0 m. Therefore, we experimentally obtained two measurement models at 1.25 m, 2.50 m used for basin conditions as shown in Table 2 and Table 3.

## 4. Experiment

### 4.1. Experimental Setup

**Figure 11:**
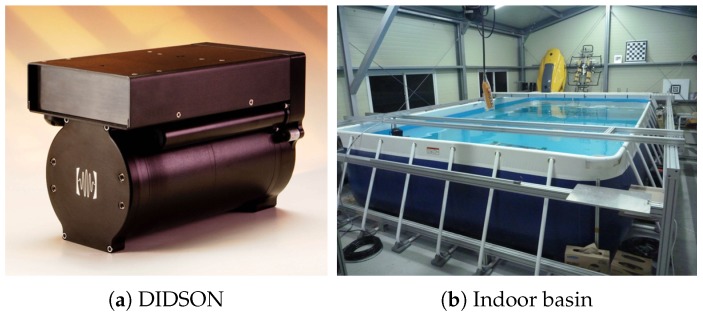
Experimental equipment.

**Figure 12:**
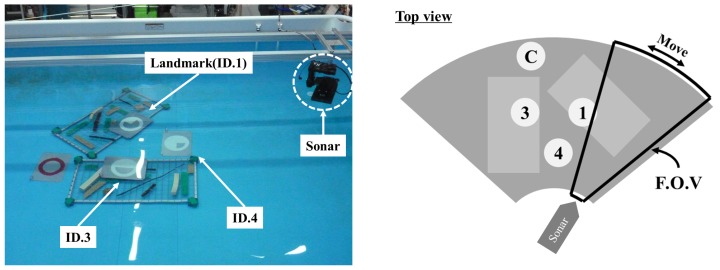
Experimental setup for multi-object recognition.

The experiments for demonstrating the performance of the proposed framework were conducted in a basin at the Korea Research Institute of Ships and Ocean engineering (KRISO) in Daejeon, Korea. As shown in Figure 11b, the basin has dimensions of 7×4.5×1.2 m (length × width × depth) and, as mentioned, we used an imaging sonar called DIDSON shown in Figure 11a [24]. A DIDSON uses a 1.8 MHz acoustic source to acquire the sonar image. As shown in Figure 12, the artificial landmarks ID.1, ID.3, and ID.4 are placed on the bottom where various objects such as a wire mesh, wood, circular fake landmark, etc. are positioned in order to simulate an underwater environment. The distances of these objects from the sonar are at least 1.7 m to 2.3 m. In addition, the sonar gazes at these objects with a pose that is tilted $20^{\circ }$ and from a distance between 1.5 m to 4.0 m.

**Figure 13:**
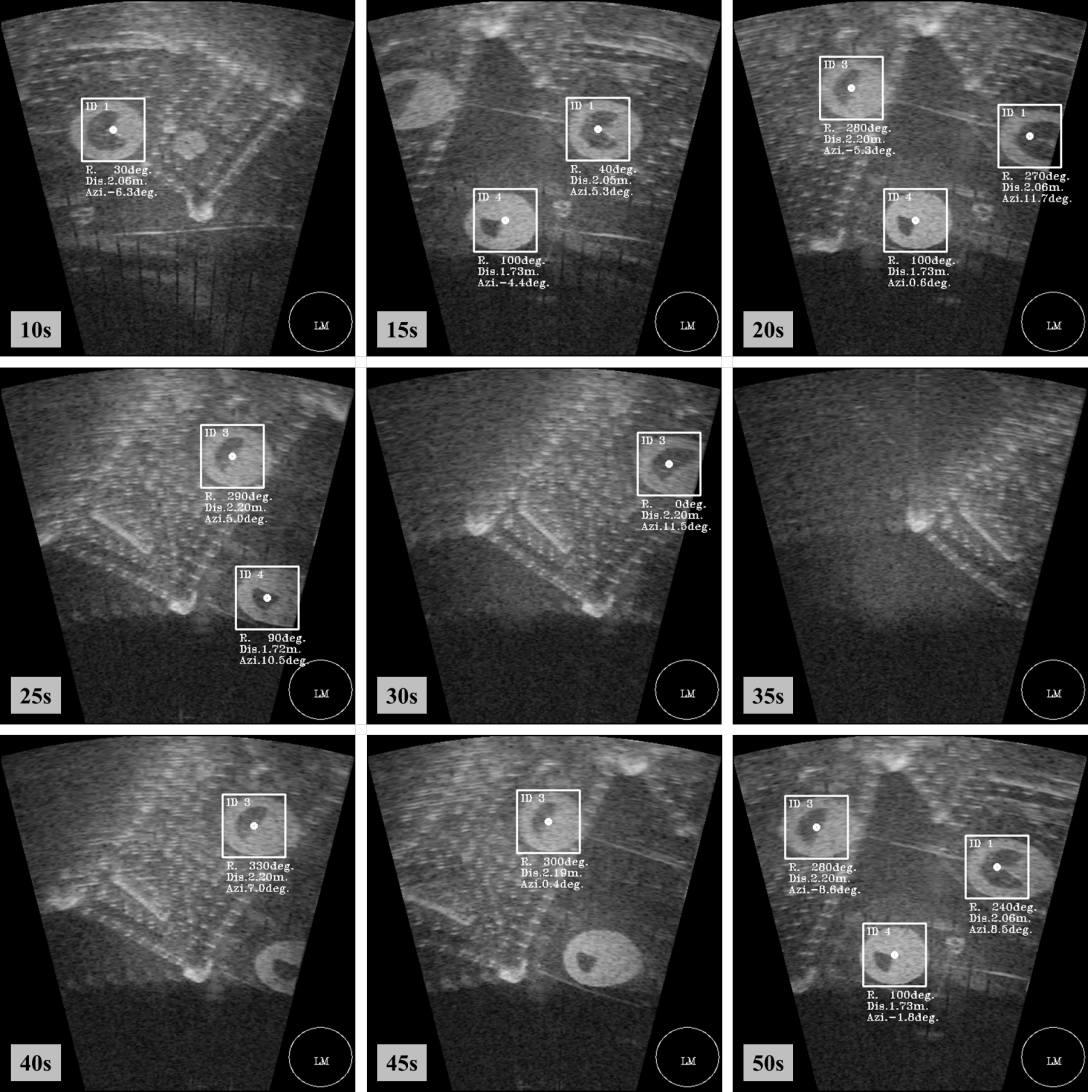
Experimental results of the proposed recognition framework method.

While the sonar rotated two times between −$40^{\circ }$ and $40^{\circ }$ at a constant speed of 2${}^{\circ }/s$, we performed real-time recognition using the proposed framework and obtained 992 successive sonar images. The qualitative images of this experiment from the at 1st rotation are shown in Figure 13.

### 4.2. Analysis

The receiver operating characteristic (ROC) analysis is adopted to evaluate the recognition performance [25]. The main indexes of performance used in this method are true positive rate (TPR), also called sensitivity and false positive rate (FPR), also called 1-specificity. In this experiment, TPR indicates that the recognition method correctly recognized the landmark’s ID and perceived the absence of landmarks. On the contrary, FPR indicates that the recognition method missed the existence and IDs of landmarks. Hence, we want to obtain results with high TPR and low FPR from this experiment. Furthermore, we also evaluate a shape matrices identification (SMI) method by ROC analysis to compare the performance. Although the SMI method has been used for update state as the measurement in the proposed framework, it is necessary to compare the performance of the SMI method using a single frame and the proposed framework using consecutive frames for recognition.

Based on the results shown in Figure 13, we present the results of the ROC analysis in Table 4 and Table 5. The proposed framework shows better recognition results with TPR of 0.8648 and FPR of 0.0276. In the case of ID.3, in the proposed framework, TPR is 0.8671 and FPR is 0.0325, which is superior to the SMI method. This is because ID.1 is often recognized as ID.3 in the SMI method. As shown in Figure 13, the shape and boundary of objects are not clear and appear to be misty due to the characteristics of sonar images, such as speckle noise and the residual image by reflection of acoustic sound. For the identification in the case of the SMI method, an edge image is required to find the center of the landmark; however, this is not easy in the case of a sonar image. Hence, the inner fan shape of the landmark is incorrectly detected by the SMI method. On the other hand, the recognition using the proposed framework method is more accurate in this case.

**Figure 14:**
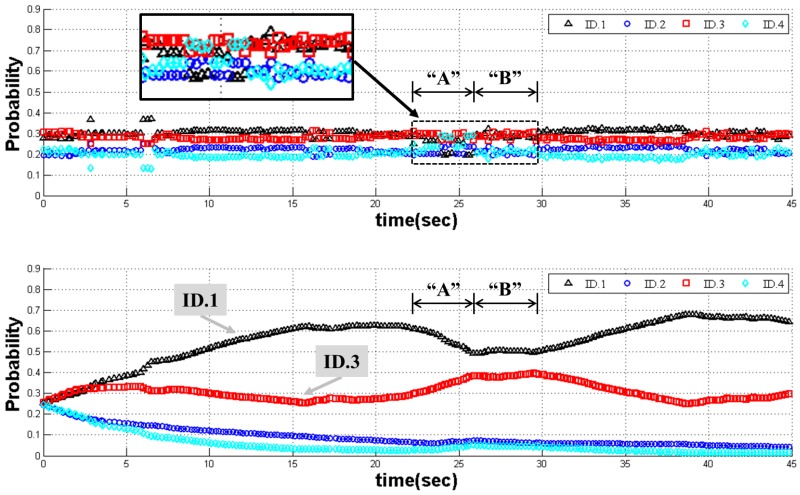
Trend line of probability ID.1: these graphs show the results of the SMI method [23] (top) and the proposed framework method (bottom).

Figure 14 shows the recognition probability of ID.1. As shown in the top of Figure 14, the SMI method does not provide salient recognition results. In particular, ID.3 has a higher probability than ID.1 in an area of “A” than the others in the SMI method. In addition, it is confused in area “B”. On the contrary, in the case of the proposed framework method, the proposed method identifies the object to be ID.1 after about 4 s. Considering that the process speed is four frames per second, 16 sonar images are used for the first recognition in this experiment. After that time, the proposed method provides correct recognition results with the highest probability of ID.1. Although the probability of ID.1 is temporarily reduced in areas “A” and “B”, the ID.1 landmark is not determined as ID.3. Thus, this result shows that the proposed framework method overcomes the problem due to the weakness of sonar image by using continuous probability evaluation.

We used a total of 992 sonar images in the experiment. As shown in Table 4, each total β is not 992 images except for ID.2. The reason for the transient duplicate detection is that the different landmarks are recognized as one of the landmarks. Hence, TP of the real landmark is increased again and FN of incorrectly recognized landmarks is increased. On the contrary, we find that the total βs are 992 images in the results of the proposed framework method in Table 5. As shown in Figure 14, the probability of ID.1 is continuously increased according to the detection in every single image. Therefore, we can find that the ID, having a large probability, is an ID of the landmark by comparison with each object and each ID.

## 5. Conclusions

In this paper, we proposed a probability-based framework for underwater object recognition using sonar images. The acoustic image received from an imaging sonar is unstable due to ultrasonic waves. Hence, it is difficult to detect and recognize objects. To solve this problem, simple-shaped artificial landmarks were designed to improve detectability by the imaging sonar. Furthermore, to detect these landmarks, we adopted a probability-based recognition framework, which consists of three steps: (1) candidate selection, (2) continuity evaluation and (3) Bayesian estimation for recognition. We selected a number of objects with high similarity scores as candidates using Hough circle transform. The continuity of candidates in a series of images is evaluated by particle filter. Lastly, a recognition method based on Bayesian rule verifies the probability of obtaining the ID of each candidate. The experiment was performed to compare the performance of the proposed framework method with that of the SMI method and the results show that the proposed framework method, with continuous probability evaluation, enhances the recognition performance in the sonar image.

By developing and verifying the proposed method, we have found some practical and challenging issues for the underwater object recognition using sonar images. First, the acoustic frequency of imaging sonar highly affects the quality of object recognition. Adequate acoustic frequency should be selected according to the application. Second, characteristics of sonar image are quite different from the optical image. Image processing should be performed by considering the characteristics of sonar image not directly using the conventional technique of optical image. Finally, it is not easy to recognize arbitrary underwater objects using imaging sonar. Thus, we need to develop a recognition method considering a target object and its surface condition that affects the amount of reflective acoustic wave.

Future plans for further improvement to the proposed method include the following: (1) the proposed method can be applied to other types of underwater objects. Even though object recognition of arbitrary underwater objects is difficult, it can be used to recognize specific underwater objects such as mines or pipelines; (2) we will attempt to use the object recognition method for autonomous underwater robot navigation. The underwater robot can explore the underwater environment by correcting its position using the recognized object.

## Figures and Tables

**Table 1:** Nomenclature.

$l_{t(i)}\left(m\right)$	the landmark *m*, at time t=t(i)
$M_{t(i)}$	number of all the landmarks recognized at time at time t=t(i)
$x_{t(i)}\left(m\right)$	random variable which represents the feature of the landmark $l_{t(i)}\left(m\right)$
$F_{feature}$	number of all the possible features a landmark can take
$f_{k}$	feature $k,k=1,2,\cdots ,F_{feature}$
$z(l_{t(i)}\left(m\right))$	measurement for the landmark $l_{t(i)}\left(m\right)$

**Table 2:** Measurement model 1 (range of view : 1.25m).

ID *k*	$P(f_{1}|f_{k})$	$P(f_{2}|f_{k})$	$P(f_{3}|f_{k})$	$P(f_{4}|f_{k})$
1	0.3195	0.2392	0.2608	0.1805
2	0.2463	0.3168	0.1834	0.2535
3	0.2554	0.1742	0.3230	0.2474
4	0.1715	0.2452	0.2548	0.3285

**Table 3:** Measurement model 2 (range of view : 2.50m).

ID *k*	$P(f_{1}|f_{k})$	$P(f_{2}|f_{k})$	$P(f_{3}|f_{k})$	$P(f_{4}|f_{k})$
1	0.3341	0.2480	0.2520	0.1659
2	0.2637	0.3087	0.1922	0.2354
3	0.2399	0.1769	0.3229	0.2603
4	0.1681	0.2485	0.2515	0.3319

**Table 4:** Recognition results of the SMI method.

Metrics	ID.1	ID.2	ID.3	ID.4	Total α
TPR (true positive rate)	0.9751	0.0000	0.7666	0.7156	0.8226
FPR (false positive rate)	0.0813	0.0030	0.0310	0.0559	0.0381
TP (true positive)	353	0	266	239	858
TN (true negative)	588	989	626	625	2828
FP (false positive)	52	3	20	37	112
FN (false negative)	9	0	81	95	185
Total β	1002	992	993	996	3983

**Table 5:** Recognition results of the proposed framework.

Metrics	ID.1	ID.2	ID.3	ID.4	Total α
TPR	0.9943	0.0000	0.8671	0.7242	0.8648
FPR	0.0453	0.0000	0.0325	0.0468	0.0276
TP	350	0	300	239	889
TN	611	992	625	631	2859
FP	29	0	21	31	81
FN	2	0	46	91	139
Total β	992	992	992	992	3968
